# Empirical Bayes Model Comparisons for Differential Methylation Analysis

**DOI:** 10.1155/2012/376706

**Published:** 2012-08-22

**Authors:** Mingxiang Teng, Yadong Wang, Seongho Kim, Lang Li, Changyu Shen, Guohua Wang, Yunlong Liu, Tim H. M. Huang, Kenneth P. Nephew, Curt Balch

**Affiliations:** ^1^School of Computer Science and Technology, Harbin Institute of Technology, Harbin 150001, China; ^2^Department of Bioinformatics and Biostatistics, University of Louisville, Louisville, KY 40292, USA; ^3^Division of Biostatistics, Department of Medicine, Indiana University School of Medicine, Indianapolis, IN 46202, USA; ^4^Center for Computational Biology and Bioinformatics, Indiana University School of Medicine, Indianapolis, IN 46202, USA; ^5^Indiana University Melvin and Bren Simon Cancer Center, Indianapolis, IN 46202, USA; ^6^Department of Molecular Medicine, University of Texas Health Science Center at San Antonio, San Antonio, TX 78229, USA; ^7^Medical Sciences Program, Indiana University School of Medicine, Bloomington, IN 47405, USA; ^8^Department of Obstetrics and Gynecology, Indiana University School of Medicine, Indianapolis, IN 46202, USA

## Abstract

A number of empirical Bayes models (each with different statistical distribution assumptions) have now been developed to analyze differential DNA methylation using high-density oligonucleotide tiling arrays. However, it remains unclear which model performs best. For example, for analysis of differentially methylated regions for conservative and functional sequence characteristics (e.g., enrichment of transcription factor-binding sites (TFBSs)), the sensitivity of such analyses, using various empirical Bayes models, remains unclear. In this paper, five empirical Bayes models were constructed, based on either a gamma distribution or a log-normal distribution, for the identification of differential methylated loci and their cell division—(1, 3, and 5) and drug-treatment-(cisplatin) dependent methylation patterns. While differential methylation patterns generated by log-normal models were enriched with numerous TFBSs, we observed almost no TFBS-enriched sequences using gamma assumption models. Statistical and biological results suggest log-normal, rather than gamma, empirical Bayes model distribution to be a highly accurate and precise method for differential methylation microarray analysis. In addition, we presented one of the log-normal models for differential methylation analysis and tested its reproducibility by simulation study. We believe this research to be the first extensive comparison of statistical modeling for the analysis of differential DNA methylation, an important biological phenomenon that precisely regulates gene transcription.

## 1. Introduction

High-density oligonucleotide tiling arrays have been widely utilized to globally analyze chromatin modifications across entire genomes, including assessments of DNA methylation, in addition to the identification of transcription factor binding sites [1–7]. Although the novel sequencing technology introduces more effective and powerful approach than tiling arrays, recently, some custom-designed tiling arrays still hold great promise of advantages, for example, cost-effectiveness and region customization. In this paper, we investigated genome-wide DNA methylation patterns, following 1, 3, and 5 cell divisions and exposure to a DNA-damaging agent (the DNA-crosslinking agent cisplatin) using differential methylation hybridization (DMH) analysis, a microarray-based, two-color hybridization [8, 9]. 

To date, there have been numerous statistical inference frameworks developed for microarray differential analysis, including empirical [10] and nonempirical Bayes [11] and frequentist approaches [12]. As the empirical Bayes model can borrow information across samples and probes, it has the advantage over the frequentist approach in small sample problems. Moreover, as compared to the nonempirical Bayes model, it does not depend on a predefined and subjective prior distribution, as it provides estimation of prior distribution and other parameters simultaneously. In the last decade, numerous empirical Bayes methods and algorithms have been applied to analyze microarray-based studies, including gene expression [13–16], protein-to-DNA binding (chromatin-immunoprecipitation (ChIP)) [17, 18], and DNA methylation [19, 20]. Therefore, in this study, we performed a comparison of the accuracy of various empirical Bayes models for analyzing these universally utilized biological assessments. 

The fundamental key of empirical Bayes model for characterizing microarray data is the statistical distribution assumption, which currently includes two common types: log-normal and gamma distribution. Our group was one of the first to use the empirical Bayes model for the analysis of differential methylation microarray data, by developing a log-normal empirical Bayes model for microarray analysis of not only differential DNA methylation but also histone acetylation and differential gene expression, in a “triple array” system for the simultaneous assessment of these phenomena in ovarian cancer cells [21]. We then developed a gamma-normal-gamma mixture model to investigate three differentially methylated loci in three breast cancer cell lines [22]. More recently, a joint log-normal empirical Bayes model was developed to investigate the correlation between gene expression and DNA methylation [19]. Although both log-normal and gamma distributions gave rational hypothesis on methylation related analysis, it is not clear which statistical distribution assumption provides the best differential methylation analysis. To date, statistical comparison between two distribution assumptions was never performed regarding differential methylation analysis.

It was recently shown that specific sequence characteristics of methylated regions exist in cancerous [23, 24] and in normal tissues [25–28]. These sequence characteristics include pattern frequencies, DNA structure prediction, CpG islands, and transcription factors in promoter regions. Our own work has shown that hypermethylated gene promoters had enriched transcription factor-binding sites (TFBSs) in ovarian cancer chemo drug-resistant cells [29] and DNA methylation fidelity is greatly attributed to *cis*-regulatory elements [30]. Since hyper- or hypomethylated CpG islands are usually chosen from differentially methylated microarray probe sequences, it is very critical to study how sensitive TFBSs are enriched among these sequences selection due to the log-normal or gamma distribution assumptions in the empirical Bayes model. Therefore, TFBS enrichment analysis could be used to provide biological evaluations of the accuracy of various models used in differential methylation microarray analysis.

In this paper, we constructed and compared the performance of a number of empirical Bayes models, based on log-normal and gamma distributions, and then compared their performance in differential methylation analysis on real data. Finally, we assessed the impact of these models for a common biological application, TFBS enrichment within DNA sequences differentially methylated by cell division and treatment with a DNA-damaging agent.

## 2. Materials and Methods

### 2.1. DNA Methylation Assessment

Genomic DNA from ovarian cancer A2780 cells (ATCC, Manassas, VA, Calbiochem, Billerica, MA, USA) and total genomic DNA purified (DNeasy purification kits, Qiagen, Valencia, CA) following 1, 3, and 5 cell divisions were exposed or unexposed to the DNA adduct-forming agent cisplatin. Differential methylation hybridization (DMH) was then performed as previously described [31–33]. Briefly, isolated DNA was digested with the methylation-insensitive restriction enzyme BfaI (C^*∧*^TAG), followed by ligation of linkers. Linker-ligated DNA was then digested by the methylation-sensitive (i.e., methylated cytosines being cleavage resistant) enzymes HinP1I (G^*∧*^CGC) and HpaII (C^*∧*^CGG), and digestion products were then amplified by linker PCR (restriction enzymes from New England Biolabs, Ipswich, MA). The PCR products were further amplified using aminoallyl-dUTP incorporation to facilitate labeling with the fluorophores Cy3 (parental A2780) or Cy5 (1, 3, and 5 divisions of A2780 cells combined with treatment with the DNA-crosslinking agent cisplatin). The labeled DNA samples were then combined and hybridized to a customized 60-mer oligo-microarray containing 40,000 CpG-rich fragments from 12,000 known gene promoters (Agilent, Santa Clara, CA). Following hybridization and washing, microarray images were scanned and generated using an Axon GenePix 4200A scanner (Molecular Devices, Sunnyvale, CA). All DMH DNA methylation data, in MIAME-compliant format, has been deposited and can be accessed using Gene Expression Omnibus (http://www.ncbi.nlm.nih.gov/projects/geo/) SuperSeries code GSE15709.

### 2.2. DNA Methylation Microarray Normalization

A numerical methylation signal for each probe, *y*, and its associated SE (background variation), *σ*, were defined as follows:

(1) y=|F−B|,  σ=SDF2PixF+SDB2PixB,  σ′=σy,

where *F* and *B* represent the foreground and background intensities of the same dye (Cy3 or Cy5), respectively, and SD and Pix are the probe signal standard deviation and pixel number for the corresponding probe, respectively. Then, a Lowess normalization was performed between M-A plots for Cy3/Cy5 probe signals for each array and for different arrays, and each probe's *σ* was rescaled according to this normalization [34]. 

### 2.3. Empirical Bayes Models

#### 2.3.1. Binary-Gamma-Gamma Model (BGG)

For our use of the BGG model, first proposed by Newton et al. [10], we assumed that any specific probe *i* in both the parental (A2780) and cisplatin-treated daughter cells had the same true but unobserved methylation signal (H_0_), *θ*_*i*_ ~ Γ(*a*_0_, *ν*) if it was not differentially methylated. We therefore, denoted (*y*_*irk*_*r*__, *y*_*igk*_*g*__) as the observed methylation signals having a between-channel and between-replicate variation described by *y*_*irk*_*r*__ ~ Γ(*a*, *θ*_*i*_) and *y*_*igk*_*g*__ ~ Γ(*a*, *θ*_*i*_), where (*r*, *g*) denote the Cy5 (cisplatin-treated A2780 progeny cells) and Cy3 (parental, untreated A2780 cells) fluorescence values and (*k*_*r*_, *k*_*g*_) represent the technical replicates. If a probe *i* was differentially methylated (H_A_), its true but unobserved methylation in cisplatin-treated daughter cells and untreated parental A2780 cells was represented by two different random variables from the same gamma distribution, *θ*_*ir*_ ~ Γ(*a*_0_, *ν*) and *θ*_*ig*_ ~ Γ(*a*_0_, *ν*), and its between-replicate variation follows the same gamma distributions as those under H_0_. With the marginal probabilities under H_0_ and H_A_ denoted as *p*_0_(*y*_*ir*_, *y*_*ig*_) and *p*_*A*_(*y*_*ir*_, *y*_*ig*_), respectively, *y*_*ir*_ = {*y*_*irk*_*r*__} and *y*_*ig*_ = {*y*_*igk*_*g*__} and their likelihood function (Table 2) is

(2) L(a,a0,ν,p)=∏i{(pA(yir,yig)∗p)zi∗(p0(yir,yig)∗(1−p))1−zi},

with parameter (*a*, *a*_0_, *ν*, *p*, *z*_*i*_) estimations performed by E-M algorithm and the E-Step:

(3) z^i=P(zi=1 ∣ yirkr,yigkg,a,a0,ν,p)=p∗pA(yir,yig)p∗pA(yir,yig)+(1−p)∗p0(yir,yig).

The initial values for (*a*, *a*_0_, *ν*, *p*) were set as (20, 0.6, 20, 0.2), thus allowing uniform input for all gamma models. Those values were selected by multiple trainings on the models for the purpose of efficient convergence.

Consequently, the M-step was

(4) p^=2+∑iz^i2∗2+n,

where *n* is the total number of probes and

(5) (a^,a^0,ν^)=argmax⁡a,a0,υ⁡ L(a,a0,ν,p),

where the parameters $\left(\hat{a},{\hat{a}}_{0},\hat{\nu }\right)$ were numerically optimized by the *R* function **nlminb** (more details of this derivation are provided in the Supplementary Material available online at doi:10.1155/2012/376706). 

#### 2.3.2. Binary-Normal-Gamma-Gamma Model (BNGG)

For our microarray differential methylation analysis, we slightly revised the BGG model, in which the between-replicate variation is modeled by truncated normal distributions, as follows: *y*_*irk*_*r*__ ~ TN(*η*_*ir*_, *τ*_*i*_^2^); *y*_*igk*_*g*__ ~ TN(*η*_*ig*_, *τ*_*i*_^2^) (see Table 2), while the other assumptions and parameters were kept the same. In the BNGG model, the gamma distribution Γ(*a*, *θ*_*i*_) aimed to accurately capture the between-channel variation, with the likelihood function *L*(*τ*_*i*_^2^, *a*, *a*_0_, *ν*, *p*) calculated in similarity to the BGG model. Likewise, the parameters (*τ*_*i*_^2^, *a*, *a*_0_, *ν*, *p*, *z*_*i*_) were also estimated through E-M algorithm, similar to the BGG model (for more details see the Supplementary Material), with the only difference being the estimations of the additional values of *τ*_*i*_^2^ (given a total of *n*). For this purpose, Hill Climbing was used to optimize these parameters for each iteration of the M-step, efficiently reducing the time-cost in function **nlminb**.

#### 2.3.3. Binary-Normal-Normal-Gamma-Gamma Model (BNNGG)

 Our BNNGG model was a further revision from the BNGG model, with the background variation (at the pixel level) added as an additional source of variation *y*_*irk*_*r*__ ~ TN(*η*_*ir*_, *σ*_*irk*_*r*__^2^ + *τ*_*i*_^2^); *y*_*igk*_*g*__ ~ TN(*η*_*ig*_, *σ*_*igk*_*g*__^2^ + *τ*_*i*_^2^), where (*σ*_*irk*_*r*__^2^, *σ*_*igk*_*g*__^2^) are known (as defined in (1)). The full model specification, as defined in Table 2, calculates the likelihood function *L*(*τ*_*i*_^2^, *a*, *a*_0_, *ν*, *p*) similarly to the BNGG model. Also, similar to the BNGG model, the parameters (*τ*_*i*_^2^, *a*, *a*_0_, *ν*, *p*, *z*_*i*_) were estimated through E-M algorithm (for more details see the Supplementary Material).

#### 2.3.4. Binary-Log-Normal-Normal Model (BLNN)

The BLNN model was first proposed by Kendziorski et al. [35] for the analysis of two-color (gene expression) microarray data and further revised by Li et al. [21] for analyzing both DNA methylation and histone acetylation. This model assumes that each probe *i* in both the drug-treated daughter and untreated A2780 parental cells has the same true (but unobserved) logarithmic methylation signal (H_0_), *η*_*i*_ ~ N(*μ*, *φ* ^2^) if it is not differentially methylated. Denote (*y*_*irk*_*r*__′, *y*_*igk*_*g*__′) as the log-transformed methylation signals. Their between-channel and between-replicate variations are described by *y*_*irk*_*r*__′ ~ N(*η*_*i*_, *τ*_*i*_^2^) and *y*_*igk*_*g*__′ ~ N(*η*_*i*_, *τ*_*i*_^2^). If probe *i* is differentially methylated (H_A_), its true but unobserved logarithmic methylation in cisplatin-treated and untreated A2780 cells is two different random variables from the same normal distribution: *η*_*ir*_ ~ N(*μ*, *φ* ^2^) and *η*_*ig*_ ~ N(*μ*, *φ* ^2^). Their between-channel and between-replicate variations follow the same normal distributions as those under H_0_. Their likelihood function *L*(*τ*_1_^2^,…, *τ*_*n*_^2^, *μ*, *φ* ^2^, *p*) is described in Table 2. Parameter estimations were performed with E-M algorithm for (*τ*_*i*_^2^, *μ*, *φ* ^2^, *p*, *z*_*i*_) as procedure similar to BNGG model. Differences included the parameters *μ*, *φ* ^2^, and *p* and the E-step, initial values being 7.8, 1.8, 0.5, set to allow rapid convergence. A more detailed derivation of this model is included in the Supplementary Material.

#### 2.3.5. Binary-Log-Normal-Normal-Normal Model (BLNNN)

Our BLNNN model was revised from the BLNN model (described above), in which the background variation at the pixel level was added as an additional source of variation: *y*_*irk*_*r*__′ ~ N(*η*_*i*_, *σ*_*irk*_*r*__′^2^ + *τ*_*i*_^2^) and *y*_*igk*_*g*__′ ~ N(*η*_*i*_, *σ*_*igk*_*g*__′^2^ + *τ*_*i*_^2^), where (*σ*_*irk*_*r*__′^2^,  *σ*_*igk*_*g*__′^2^) are known and defined in (1). The full model specification is defined in Table 2. The likelihood and parameters were estimated through E-M algorithm as the same as BLNN model (for more details, see the Supplementary Material).

### 2.4. Transcription Factor-Binding Site Enrichment Analysis

Our previous study of the fidelity of DNA methylation inheritance [30] was based on the widely accepted “stochastic” DNA methylation model that predicts that the average methylation levels of specific regions result from the efficiency of two cooperative stochastic processes: heritable maintenance methylation and *de novo* methylation, occurring in concert with DNA replication [36, 37]. Consequently, in that previous analysis, we used Bayesian empirical modelling to subcategorize two subclasses showing progressive fluctuation, *stochastic hypermethylation* and *stochastic hypomethylation* (Table 1). In addition, we also observed methylated loci showing *random methylation*, defined as loci having transgenerational methylation propagation [36–38]. We then used the transcription factor-binding site (TFBS) search tool MATCH [39], a weight matrix-based software, to predict TFBSs based on the DNA nucleotide sequences of each microarray probe locus. Following compilation of that list of TFBSs, we determined the frequencies of the predicted TFBSs between three sequence categories of DNA methylation fidelity of inheritance (Table 3) and background sequences by Fisher's exact test, and a Bonferroni correction was implemented to justify 459 human TFBSs [40], and an individual *P* value threshold was chosen as 0.05/459/3 = 3.63*e* − 05 for multiple comparisons. The background sequences were 10000 randomly generated promoter sequences with equal length and GC component matched to the three sequence categories of DNA methylation fidelity of inheritance, as we have described previously [30].

## 3. Results and Discussion

### 3.1. Comparing the Performance of Five Empirical Bayes Models in Differential Methylation Data Analysis

As we mentioned in Section 1, we focused on empirical Bayes models in this paper because of its strength of analysing small sample size microarray studies. Our goal was to seek out a more appropriate distribution assumption and consequentially, a better model within empirical Bayes frameworks.

#### 3.1.1. Model Specifications

For identifying DNA sequences differentially methylated over 1, 3, or 5 cell divisions and/or treatment with the DNA-damaging agent cisplatin, we used a customized 60-mer oligo-two-color microarray, containing over 40,000 CpG-rich fragments from 12,000 promoters. Methylated versus unmethylated DNA fragments were separated by digesting DNA isolated from drug-treated daughter (Cy5 labeled for cell generations 1, 3, and 5) cells and untreated parental (Cy3 labeled) cells to methylation-sensitive restriction enzyme cleavage, where the raw values of each scanned fluorescent probe was preprocessed for foreground/background signal normalization, pixel number, and signal standard deviations. The raw data was first statistically normalized using the common Lowess method (see Section 2), and the five empirical Bayes models were then constructed based on their specific distributions (log-normal versus gamma) and variation sources (between-replicate, between-channel, and background variation) to classify differentially methylated probes for downstream analysis (transcription factor-binding enrichment). As described in Section 2, the five models were binary-gamma-gamma (BGG), binary-normal-gamma-gamma (BNGG), binary-normal-normal-gamma-gamma (BNNGG), binary-log-normal-normal (BLNN), and binary-log-normal-normal-normal (BLNNN) models. The distribution definition used for each model, in addition to their log likelihoods, is specified in Tables 1 and 2 (with further description in Section 2). More detailed statistical estimation algorithms (i.e., expectation-maximization (EM) algorithms) are included in Supplementary Material. Finally, EM iterations were performed until the convergences occurred with no more than 0.01% changes in the log-likelihoods.

#### 3.1.2. Differential Methylation Analysis

**Figure 1:**
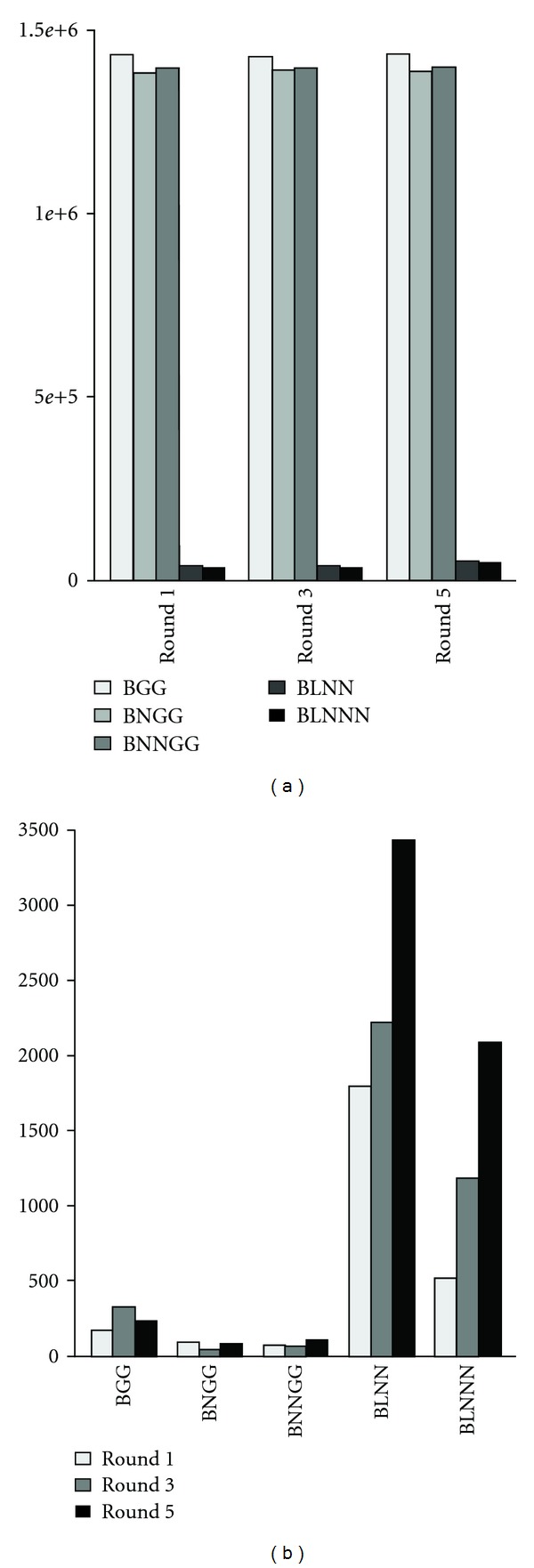
Model performance comparisons in differential methylation data analysis. Five empirical Bayes models were compared: (1) binary-gamma-gamma (BGG); (2) binary-normal-gamma-gamma (BNGG); (3) binary-normal-normal-gamma-gamma (BNNGG); (4) binary-log-normal-normal (BLNN); (5) binary-log-normal-normal-normal (BLNNN). Negative log-likelihoods (a) and the number of identified differentially methylated CpG islands (b) of the five Bayesian models as applied for comparing methylation differences between A2780 parental cells and their cisplatin-treated 1st, 3rd, and 5th generation daughter cells.

**Figure 2:**
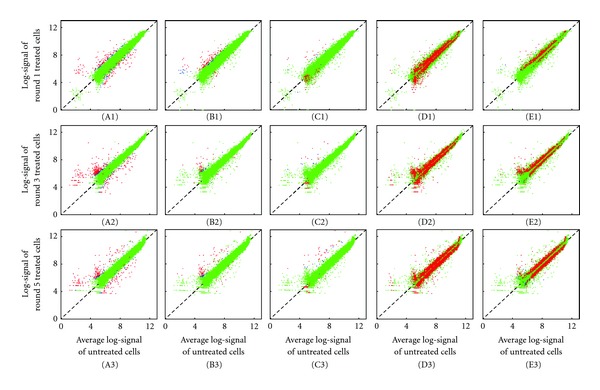
Differentially methylated CpG islands before and after cisplatin treatment identified by empirical Bayes models. Scatter plots of the logarithmically transformed DNA methylation intensities before and after 1, 3, and 5 cell divisions of cisplatin-treated A2780 cells, in which the *x*-axis represents the parental A2780 cell line and the *y*-axis represents the cisplatin-treated A2780 progeny sublines. Rows 1, 2, and 3 represent the A2780 sublines following 1, 3, and 5 cell divisions coincident to treatment with the DNA crosslinking agent cisplatin. Columns 1, 2, 3, 4, and 5 represent binary-gamma-gamma (BGG) model, binary-normal-gamma-gamma (BNGG) model, binary-normal-normal-gamma-gamma (BNNGG) model, binary-log-normal-normal (BLNN) model, and binary-log-normal-normal-normal (BLNNN) model, respectively. Red, blue, and green represent the differentially methylated CpG islands (*Z*_*i*_⩾0.8), not determined differentially methylated or not (0.2 < *Z*_*i*_ < 0.8) and not differentially methylated CpG islands (*Z*_*i*_ ⩽ 0.2).

Each of the five empirical Bayes models was then compared for its performance, as determined by the minimized negative after-convergence log-likelihoods for the EM iterations (Figure 1(a)) for the distributions of the differentially methylated probes after cell divisions for 1, 3, and 5 generations (see Section 2). It is clear that the BLNN/BLNNN models outperformed the BGG/BNGG/BNNGG models, with significantly lower negative log-likelihoods (on average, 4.26*e* + 04/4.14*e* + 04 versus 1.44*e* + 06/1.39*e* + 06/1.40*e* + 06), suggesting that log-normal is more accurate than gamma distributions in modelling microarray-based differential methylation data. However, given the log-normal model assumption, BLNNN performed better than the BLNN model, likely due to its ability to consider variations in the methylation probe level backgrounds (noise). Quantitatively, the BGG/BNGG/BNGG models identified less than 400 loci (Figure 1(b)) having differential methylation after three cisplatin-treated A2780 cell generations, while also showing no consistent patterns among the 3 models. In addition, the BGG/BNGG/BNGG models seemed applicable only to loci having noticeable differences in their observed methylation signals, thus neglecting the various variation sources (Figure 2), and, consequently, provided no benefits over the empirical Bayes model. Conversely, both the BLNN and BLNNN models showed consistently increasing number of differentially methylated loci from round 1 to 5, in accord with previous studies by our group [41] and others showing cisplatin-associated *de novo *methylation. Interestingly, BLNNN yielded less differentially methylated loci than BLNN (Figure 1(b)), likely due to low signals and oversensitivity to probe level background noise (Figure 2), thus indicating the importance of considering background noise when identifying differentially methylated loci and the better performance of BLNNN model. 

### 3.2. Transcription Factor Enrichment Analysis of Stochastic Differential Methylation Probes

#### 3.2.1. Time Dependent Differential Methylation Patterns

**Figure 3:**
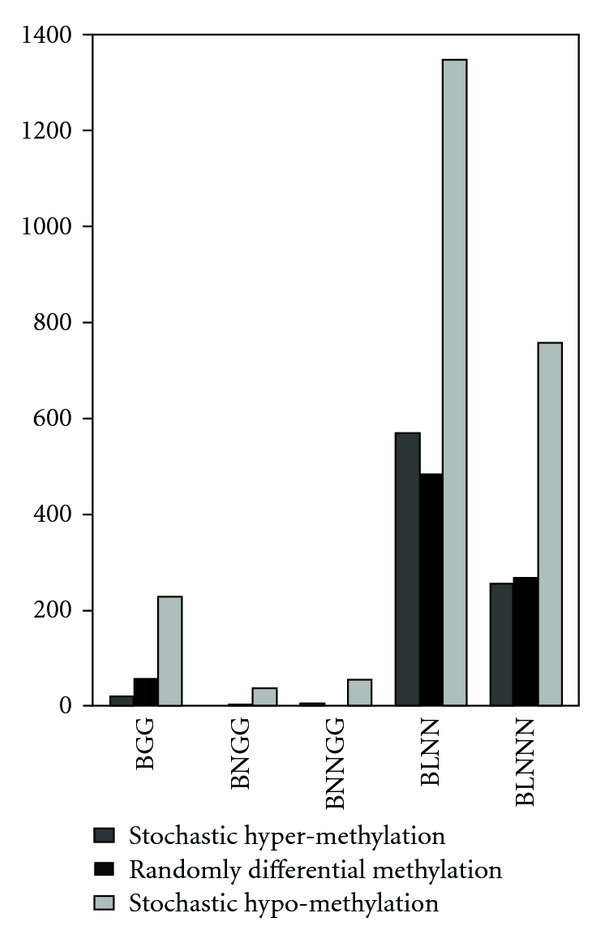
Numbers of CpG islands, as identified by empirical Bayes models, segregating into our three previously defined methylation heritability categories [30].

**Figure 4:**
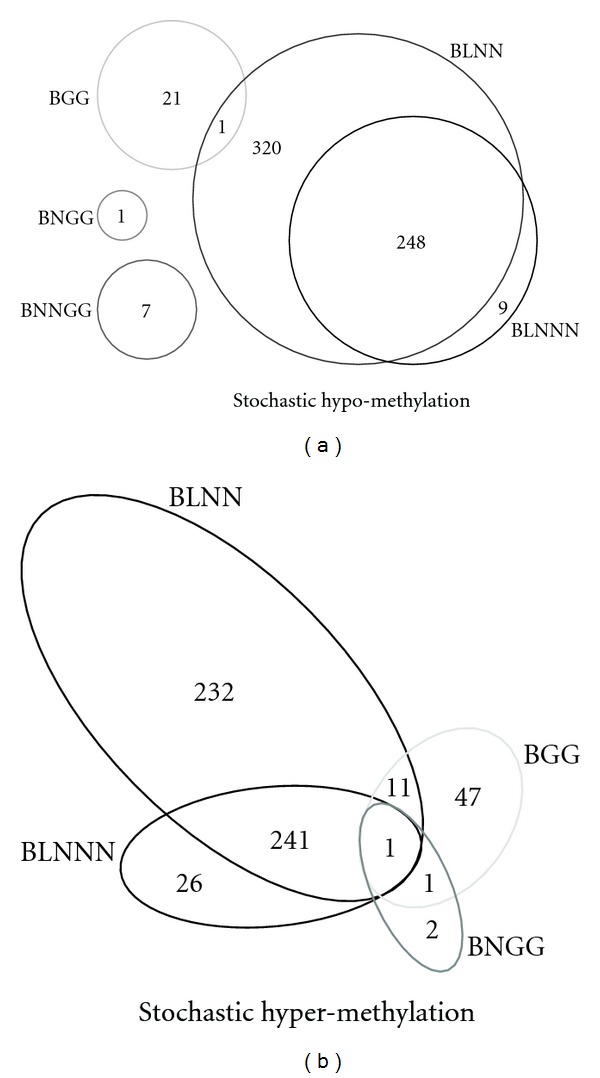
Overlaps of stochastically hypo- and hypermethylated CpG islands identified by empirical Bayes models.

Prescribed differential methylation analysis is applicable to compare DNA methylation signals before and after A2780 cells divided and were treated with cisplatin at a given time point. Our previous study of the heritable fidelity of DNA methylation during DNA replication [30], based on the widely accepted “stochastic” DNA methylation model [36, 37], used Bayesian empirical modelling to subcategorize two subclasses showing *stochastic hypermethylation* (progressively increased) and *stochastic hypomethylation* (progressive decreased), showed distinct cell division and DNA damage effect on alterations in methylation patterns [30]. To summarize the cell division-dependent differential methylation patterns after 1, 3, and 5 A2780 cell generations, we defined three categories (Table 3) as our previous work [30]: stochastic hypomethylation describes the decreasing methylation pattern, stochastic hypermethylation describes the increasing methylation pattern, and randomly differential methylation represents nonunidirectional (or nonmonotone) methylation change from round 1 to 5. Consequently, we compared the performance of the five empirical Bayes models for correctly categorizing differentially methylated loci into the three heritability categories (Figure 3). One common feature among all five models was that random differential methylation was predominant, while in both the BLNN and BLNNN models, *stochastic hypermethylation *and *stochastic hypomethylation* yielded similar numbers of loci. We also observed numerous overlapped stochastically hypomethylated loci and hypermethylated loci among the five empirical Bayesian models (Figure 4). Among the three gamma models, however, there was little or no overlap, similar to negligible overlap between the gamma and log-normal models (Figure 4). By contrast, the two log-normal models showed considerable overlap of methylation patterns within the two methylation heritability categories, although slightly more loci were identified by the BLNN.

#### 3.2.2. Transcription Factor-Binding Site (TFBS) Enrichment Analysis

To assess a possible systems biological application for this work, we compared the degree of TFBS enrichment among stochastically hypomethylated, stochastically hypermethylated, and randomly differentially methylated loci, as compared to the predicted TFBS frequencies calculated from the GC content-matched background sequences (see Section 2 for more details). Of the five models, BLNN yielded 71 TFBSs enriched in the stochastically hypomethylated loci, 51 enriched TFBSs in the stochastically hypermethylated loci, and 19 enriched TFBSs in the randomly differentially methylated loci, as compared to the background sequences (Table 4). BLNNN had very similar TFBS enrichment analyses in the stochastic hypomethylation and hypermethylation categories, with 36 and 58 enriched TFBSs, respectively. However, BLNNN had 0 enriched TFBS in the randomly differentially methylated loci, while the gamma models had essentially no enriched TFBSs in all three methylation categories (although BGG categorized four enriched TFBSs among randomly differentially methylated loci). These results indicate that TFBS enrichment analysis is highly sensitive to the empirical Bayes model distribution assumption and that stochastically differentially methylated loci selected by log-normal models are more sensitive for TFBS enrichment, as compared to the gamma models.

### 3.3. Biological Justification for the Suitability of BLNNN Model

The log-normal models presented minimum negative log-likelihoods, showing consistently increasing numbers of differential methylated and reasonable numbers of time-dependent differentially methylated loci. All these features suggest rigorous statistical performance on differential methylation analysis. Moreover, we recently found that hypermethylated gene promoters had enriched transcription factor-binding sites (TFBSs) in ovarian cancer drug-resistant cells [29] and that DNA methylation fidelity is strongly influenced by the presence of *cis*-regulatory elements [30], thus allowing differential methylation identification to have considerable biological significance. Basically, the rule of TFBS enrichment in regulating DNA methylation is that TFBSs should be enriched at promoter sequences where DNA methylation (stochastic hypo- or hyper-methylation) plays a critical role in regulating gene expression in both normal and cancer cells, with little or no enrichment at sequences where DNA methylation (random or nonmonotone) has minimal biological function [30]. By utilizing the same experiment sets and same methods to calculate TFBS enrichments (Table 4) as in our previous study [30], we found no TFBS enrichment in stochastic hypo- or hypermethylation by gamma models, which indicates the inaccurate identification on differential methylation. On the contrary, log-normal models provide biological meaningful results. Again, this suggests a better applicability of log-normal distribution assumption on differential methylation analysis. 

As we discussed previously, BLNN performed worse on low signal probes than BLNNN, which resulted in more differential methylated loci. Subsequently, BLNN generated more stochastically hypomethylated loci, stochastically hypermethylated loci, and random differential methylated loci. TFBS enrichment showed similar patterns on stochastic hypo- or hypermethylation between both models, while dramatically different on random methylation, which gives us a chance to compare these two models biologically. By enrichment of 0 versus 19, BLNNN selected purely the nonmonotone methylation loci into the random methylation pattern, suggesting a better performance than BLNN.

### 3.4. The Reproducibility of Log-Normal Model on Simulation Studies

To illustrate the applicability of log-normal distribution assumption and BLNNN model in differential methylation analysis, which is not just limited in the real microarray experiments presented in this paper, we further performed simulation studies on BLNNN model. The parameter estimates (*μ*, *φ*, *τ*, *σ*′) by BLNNN of the real microarray experiments were used for data simulation, and 10% (*p*) of the probes were chosen as differentially methylated. In detail, 10000 probes were simulated with mean of *μ* and standard deviation of *φ*. Then, each probe took 3 replicates under control or treatment conditions with between-replicate variation, *τ*, and pixel level variation, *σ*′, which were added to generate the log-transformed methylation signal, *y*′. In total, 1000 iterations of data sets were simulated and inferred by BLNNN. The true positive rate and false positive rate for differentially methylated loci were averaged as 92% and 1.8%, respectively, which strongly suggests the reproducibility of differential methylation analysis by BLNNN model. 

## 4. Conclusions

We believe this is the first comparison of empirical Bayes models for analyzing differential methylation microarray data, demonstrating that log-normal distribution is statistically superior to gamma distributions. We also showed that probe level background noise can markedly confound the identification of differentially methylated loci and particularly, affect BLNN detection of loci having small methylation signals, as compared to BLNNN. In a similar study, Kendziorski et al. also compared log-normal and gamma models on differential gene expression microarray, reporting comparable performance between the two models in both data analysis and simulations [35]. One possible interpretation is that Affymetrix gene expression data fits well to either model. However, our current data, using a two-color array system, appears better suited for log-normal distribution, based on our data analysis comparisons.

In this paper, we compared all five empirical Bayes models for revealing enrichment of TFBS motifs into three distinct methylation heritability categories. While both log-normal models provided similar numbers of enriched TFBSs in stochastically hypermethylated and hypomethylated loci, all gamma models yielded only limited or no TFBSs. In the field of epigenetics, it has been hypothesized that there exist methylation-prone and methylation-resistant sequences in cancerous [23, 24] and in normal tissues [25–28], and we have now demonstrated that many of these sequences are potential TFBSs [29, 30]. This concept has been validated using laboratory-based techniques such as transcription factor-based ChIP-chip and ChIP-seq data [42, 43]. All these publications support log-normal models to provide more accurate information necessary for the study of epigenetic modifications in development, homeostasis, and disease. 

## Figures and Tables

**Table 1:** Five empirical Bayes models parameter list.

Empirical Bayes model	Parameters	Observed data	Missing data
BGG	*a*, *a*_0_, *ν*, *p*	*y*_*ijk*_	*z*_*i*_
BNGG	*a*, *a*_0_, *ν*, *τ*_*i*_, *p*	*y*_*ijk*_	*z*_*i*_
BNNGG	*a*, *a*_0_, *ν*, *τ*_*i*_, *p*	*y*_*ijk*_, *σ*_*ijk*_	*z*_*i*_
BLNN	*μ*, *φ*, *τ*_*i*_, *p*	*y*_*ijk*_′	*z*_*i*_
BLNNN	*μ*, *φ*, *τ*_*i*_, *p*	*y*_*ijk*_′, *σ*_*ijk*_′	*z*_*i*_

Note: *i*, *j* and *k* represent probe, sample and replicate, respectively.

**Table 2:** Five empirical Bayes model frameworks.

Empirical Bayes model	H_0_ : *p*_0_(*y*_*ir*_, *y*_*ig*_)	H_A_ : *p*_*A*_(*y*_*ir*_, *y*_*ig*_)	Likelihood ∏_*i*_{(*p*_*A*_(*y*_*ir*_, *y*_*ig*_)∗*p*)^*z*_*i*_^∗(*p*_0_(*y*_*ir*_, *y*_*ig*_)∗(1 − *p*))^1−*z*_*i*_^}
	H_0_ : *θ*_*ir*_ = *θ*_*ig*_ = *θ*_*i*_ (*r* ≠ *g*)	H_A_ : *θ*_*ir*_ ≠ *θ*_*ig*_ (*r* ≠ *g*)	
BGG	*y*_*irk*_*r*__ ~ Γ(*a*, *θ*_*i*_); *y*_*igk*_*g*__ ~ Γ(*a*, *θ*_*i*_)	*y*_*irk*_*r*__ ~ Γ(*a*, *θ*_*ir*_); *y*_*igk*_*g*__ ~ Γ(*a*, *θ*_*ig*_)	*L*(*a*, *a*_0_, *ν*, *p*)
	*θ*_*i*_ ~ Γ(*a*_0_, *ν*)	*θ*_*ir*_ ~ Γ(*a*_0_, *ν*); *θ*_*ig*_ ~ Γ(*a*_0_, *ν*)	

	H_0_ : *θ*_*ir*_ = *θ*_*ig*_ = *θ*_*i*_ (*r* ≠ *g*)	H_A_ : *θ*_*ir*_ ≠ *θ*_*ig*_ (*r* ≠ *g*)	
	*y*_*irk*_*r*__ ~ TN(*η*_*ir*_, *τ*_*i*_^2^)	*y*_*irk*_*r*__ ~ TN(*η*_*ir*_, *τ*_*i*_^2^)	
BNGG	*y*_*igk*_*g*__ ~ TN(*η*_*ig*_, *τ*_*i*_^2^)	*y*_*igk*_*g*__ ~ TN(*η*_*ig*_, *τ*_*i*_^2^)	*L*(*τ*_1_^2^,…, *τ*_*n*_^2^, *a*, *a*_0_, *ν*, *p*)
	*η*_*ir*_ ~ Γ(*a*, *θ*_*i*_); *η*_*ig*_ ~ Γ(*a*, *θ*_*i*_)	*η*_*ir*_ ~ Γ(*a*, *θ*_*ir*_); *η*_*ig*_ ~ Γ(*a*, *θ*_*ig*_)	
	*θ*_*i*_ ~ Γ(*a*_0_, *ν*)	*θ*_*ir*_ ~ Γ(*a*_0_, *ν*); *θ*_*ig*_ ~ Γ(*a*_0_, *ν*)	

	H_0_ : *θ*_*ir*_ = *θ*_*ig*_ = *θ*_*i*_ (*r* ≠ *g*)	H_A_ : *θ*_*ir*_ ≠ *θ*_*ig*_ (*r* ≠ *g*)	
	*y*_*irk*_*r*__ ~ TN(*η*_*ir*_, *σ*_*irk*_*r*__^2^ + *τ*_*i*_^2^)	*y*_*irk*_*r*__ ~ TN(*η*_*ir*_, *σ*_*irk*_*r*__^2^ + *τ*_*i*_^2^)	
BNNGG	*y*_*igk*_*g*__ ~ TN(*η*_*ig*_, *σ*_*igk*_*g*__^2^ + *τ*_*i*_^2^)	*y*_*igk*_*g*__ ~ TN(*η*_*ig*_, *σ*_*igk*_*g*__^2^ + *τ*_*i*_^2^)	*L*(*τ*_1_^2^,…, *τ*_*n*_^2^, *a*, *a*_0_, *ν*, *p*)
	*η*_*ir*_ ~ Γ(*a*, *θ*_*i*_); *η*_*ig*_ ~ Γ(*a*, *θ*_*i*_)	*η*_*ir*_ ~ Γ(*a*, *θ*_*ir*_); *η*_*ig*_ ~ Γ(*a*, *θ*_*ig*_)	
	*θ*_*i*_ ~ Γ(*a*_0_, *ν*)	*θ*_*ir*_ ~ Γ(*a*_0_, *ν*); *θ*_*ig*_ ~ Γ(*a*_0_, *ν*)	

	H_0_ : *η*_*ir*_ = *η*_*ig*_ = *η*_*i*_ (*r* ≠ *g*)	H_A_ : *η*_*ir*_ ≠ *η*_*ig*_ (*r* ≠ *g*)	
BLNN	*y*_*irk*_*r*__′ ~ *N*(*η*_*i*_, *τ*_*i*_^2^); *y*_*igk*_*g*__′ ~ *N*(*η*_*i*_, *τ*_*i*_^2^)	*y*_*irk*_*r*__′ ~ *N*(*η*_*ir*_, *τ*_*i*_^2^); *y*_*igk*_*g*__′ ~ *N*(*η*_*ig*_, *τ*_*i*_^2^)	*L*(*τ*_1_^2^,…, *τ*_*n*_^2^, *μ*, *φ* ^2^, *p*)
	*η*_*i*_ ~ *N*(*μ*, *φ* ^2^)	*η*_*ir*_ ~ *N*(*μ*, *φ* ^2^); *η*_*ig*_ ~ *N*(*μ*, *φ* ^2^)	

BLNNN	H_0_ : *η*_*ir*_ = *η*_*ig*_ = *η*_*i*_ (*r* ≠ *g*)	H_A_ : *η*_*ir*_ ≠ *η*_*ig*_ (*r* ≠ *g*)	
	*y*_*irk*_*r*__′ ~ *N*(*η*_*i*_, *σ*_*irk*_*r*__′^2^ + *τ*_*i*_^2^)	*y*_*irk*_*r*__′ ~ *N*(*η*_*ir*_, *σ*_*irk*_*r*__′^2^ + *τ*_*i*_^2^)	
	*y*_*igk*_*g*__′ ~ *N*(*η*_*i*_, *σ*_*igk*_*g*__′^2^ + *τ*_*i*_^2^)	*y*_*igk*_*g*__′ ~ *N*(*η*_*ig*_, *σ*_*igk*_*g*__′^2^ + *τ*_*i*_^2^)	*L*(*τ*_1_^2^,…, *τ*_*n*_^2^, *μ*, *φ* ^2^, *p*)
	*η*_*i*_ ~ *N*(*μ*, *φ* ^2^)	*η*_*ir*_ ~ *N*(*μ*, *φ* ^2^); *η*_*ig*_ ~ *N*(*μ*, *φ* ^2^)	

**Table 3:** Time-dependent methylation pattern definitions. Between the parent A2780 cell and its cisplatin-treated 1st, 3rd, and 5th generation daughter cells, a probe with increased methylation (probability ≥ 0.8) is defined as hypermethylation (i.e., up), a probe with decreased methylation (probability ≥ 0.8) is defined as hypomethylation (i.e., down), and otherwise, the methylation change is even. Probes showing decreased methylation from generations 1 to 3 to 5 were defined as having “stochastic hypomethylation.” Analogously, probes showing increased methylation from generations 1 to 3 to 5 were considered to exhibit “stochastic hypermethylation.” Finally, probes showing mixed increased and decreased methylation from generations 1 to 3 to 5 were defined as having “random differential methylation.”

Categories	Differential methylation		
	Parental versus Generation 1	Parental versus generation 3	Parental versus generation 5
Stochastic hypomethylation	Down	Down	Down
	Even	Down	Down
	Even	Even	Down

Stochastic hypermethylation	Up	Up	Up
	Even	Up	Up
	Even	Even	Up

Random differential methylation	Down	Up	Down
	Down	Up	Even
	Down	Even	Down
	Even	Up	Down
	Even	Up	Even
	Even	Down	Even
	Even	Down	Up
	Up	Down	Up
	Up	Down	Even
	Up	Even	Up

**Table 4:** Number of significantly enriched TFBSs in time-dependent methylation patterns.

Empirical Bayes model	Stochastic hypo-methylation	Stochastic hyper-methylation	Random differential methylation
BGG	0	0	4
BNGG	0	0	0
BNNGG	0	0	0
BLNN	71	51	19
BLNNN	36	58	0

## Abbreviations

TFBS: Transcription factor-binding site
DMH: Differential methylation hybridization
BGG: Binary-gamma-gamma model
BNGG: Binary-normal-gamma-gamma model
BNNGG: Binary-normal-normal-gamma-gamma model
BLNN: Binary-log-normal-normal model
BLNNN: Binary-log-normal-normal-normal model
EM: Expectation-maximization.

## References

[1] Mockler TC, Ecker JR (2005). Applications of DNA tiling arrays for whole-genome analysis. *Genomics*.

[2] Liu XS (2007). Getting started in tiling microarray analysis. *PLoS Computational Biology*.

[3] Yazaki J, Gregory BD, Ecker JR (2007). Mapping the genome landscape using tiling array technology. *Current Opinion in Plant Biology*.

[4] Gendrel AV, Lippman Z, Martienssen R, Colot V (2005). Profiling histone modification patterns in plants using genomic tiling microarrays. *Nature Methods*.

[5] Zhang X, Yazaki J, Sundaresan A (2006). Genome-wide high-resolution mapping and functional analysis of DNA methylation in arabidopsis. *Cell*.

[6] Weber M, Hellmann I, Stadler MB (2007). Distribution, silencing potential and evolutionary impact of promoter DNA methylation in the human genome. *Nature Genetics*.

[7] Euskirchen GM, Rozowsky JS, Wei CL (2007). Mapping of transcription factor binding regions in mammalian cells by ChIP: comparison of array- and sequencing-based technologies. *Genome Research*.

[8] Balch C, Yan P, Craft T (2005). Antimitogenic and chemosensitizing effects of the methylation inhibitor zebularine in ovarian cancer. *Molecular Cancer Therapeutics*.

[9] Fan M, Yan PS, Hartman-Frey C (2006). Diverse gene expression and DNA methylation profiles correlate with differential adaptation of breast cancer cells to the antiestrogens tamoxifen and fulvestrant. *Cancer Research*.

[10] Newton MA, Kendziorski CM, Richmond CS, Blattner FR, Tsui KW (2001). On differential variability of expression ratios: improving statistical inference about gene expression changes from microarray data. *Journal of Computational Biology*.

[11] Parmigiani G, Garrett ES, Anbazhagan R, Gabrielson E (2002). A statistical framework for expression-based molecular classification in cancer. *Journal of the Royal Statistical Society. Series B*.

[12] Efron B (2004). Large-scale simultaneous hypothesis testing: the choice of a null hypothesis. *Journal of the American Statistical Association*.

[13] Smyth GK (2004). Linear models and empirical bayes methods for assessing differential expression in microarray experiments. *Statistical Applications in Genetics and Molecular Biology*.

[14] Johnson WE, Li C, Rabinovic A (2007). Adjusting batch effects in microarray expression data using empirical Bayes methods. *Biostatistics*.

[15] Efron B (2008). Microarrays, empirical bayes and the two-groups model. *Statistical Science*.

[16] Lo K, Gottardo R (2007). Flexible empirical Bayes models for differential gene expression. *Bioinformatics*.

[17] Keleş S (2007). Mixture modeling for genome-wide localization of transcription factors. *Biometrics*.

[18] Gottardo R, Li W, Johnson WE, Liu XS (2008). A flexible and powerful Bayesian hierarchical model for ChIP-chip experiments. *Biometrics*.

[19] Jeong J, Li L, Liu Y, Nephew KP, Huang THM, Shen C (2010). An empirical Bayes model for gene expression and methylation profiles in antiestrogen resistant breast cancer. *BMC Medical Genomics*.

[20] Aryee MJ, Wu Z, Ladd-Acosta C (2011). Accurate genome-scale percentage DNA methylation estimates from microarray data. *Biostatistics*.

[21] Li L, Shi H, Yiannoutsos C, Huang THM, Nephew KP (2005). Epigenetic hypothesis tests for methylation and acetylation in a triple microarray system. *Journal of Computational Biology*.

[22] Khalili A, Potter D, Yan P (2007). Gamma-normal-gamma mixture model for detecting differentially methylated loci in three breast cancer cell lines. *Cancer Informatics*.

[23] Goh L, Murphy SK, Muhkerjee S, Furey TS (2007). Genomic sweeping for hypermethylated genes. *Bioinformatics*.

[24] Keshet I, Schlesinger Y, Farkash S (2006). Evidence for an instructive mechanism of de novo methylation in cancer cells. *Nature Genetics*.

[25] Bock C, Paulsen M, Tierling S, Mikeska T, Lengauer T, Walter J (2006). CpG island methylation in human lymphocytes is highly correlated with DNA sequence, repeats, and predicted DNA structure. *PLoS genetics*.

[26] Bock C, Walter J, Paulsen M, Lengauer T (2007). CpG island mapping by epigenome prediction. *PLoS Computational Biology*.

[27] Das R, Dimitrova N, Xuan Z (2006). Computational prediction of methylation status in human genomic sequences. *Proceedings of the National Academy of Sciences of the United States of America*.

[28] Fang F, Fan S, Zhang X, Zhang MQ (2006). Predicting methylation status of CpG islands in the human brain. *Bioinformatics*.

[29] Li M, Paik HIH, Balch C (2008). Enriched transcription factor binding sites in hypermethylated gene promoters in drug resistant cancer cells. *Bioinformatics*.

[30] Teng M (2012). The influence of cis-regulatory elements on DNA methylation fidelity. *PLoS One*.

[31] Wei SH (2006). Prognostic DNA methylation biomarkers in ovarian cancer. *Clinical Cancer Research*.

[32] Yan PS, Wei SH, Huang TH (2002). Differential methylation hybridization using CpG island arrays. *Methods in Molecular Biology*.

[33] Yan PS (2002). Applications of CpG island microarrays for high-throughput analysis of DNA methylation. *Journal of Nutrition*.

[34] Yang YH, Dudoit S, Luu P (2002). Normalization for cDNA microarray data: a robust composite method addressing single and multiple slide systematic variation. *Nucleic acids research*.

[35] Kendziorski CM, Newton MA, Lan H, Gould MN (2003). On parametric empirical Bayes methods for comparing multiple groups using replicated gene expression profiles. *Statistics in Medicine*.

[36] Jones PA, Liang G (2009). Rethinking how DNA methylation patterns are maintained. *Nature Reviews Genetics*.

[37] Riggs AD, Xiong Z (2004). Methylation and epigenetic fidelity. *Proceedings of the National Academy of Sciences of the United States of America*.

[38] Chen ZX, Riggs AD (2005). Maintenance and regulation of DNA methylation patterns in mammals. *Biochemistry and Cell Biology*.

[39] Kel AE, Gößling E, Reuter I, Cheremushkin E, Kel-Margoulis OV, Wingender E (2003). MATCH: a tool for searching transcription factor binding sites in DNA sequences. *Nucleic Acids Research*.

[40] Strassburger K, Bretz F (2008). Compatible simultaneous lower confidence bounds for the Holm procedure and other Bonferroni-based closed tests. *Statistics in Medicine*.

[41] Li M, Balch C, Montgomery JS (2009). Integrated analysis of DNA methylation and gene expression reveals specific signaling pathways associated with platinum resistance in ovarian cancer. *BMC Medical Genomics*.

[42] Murphy DM, Buckley PG, Bryan K (2009). Global MYCN transcription factor binding analysis in neuroblastoma reveals association with distinct E-box motifs and regions of DNA hypermethylation. *PLoS ONE*.

[43] Gebhard C, Benner C, Ehrich M (2010). General transcription factor binding at CpG islands in normal cells correlates with resistance to de novo DNA methylation in cancer cells. *Cancer Research*.

